# Proper Angle of Sono-guided Central Venous Line Insertion

**Published:** 2016

**Authors:** Hassan Barzegari, Arash Forouzan, Mohammad Ali Fahimi, Behzad Zohrevandi, Mandana Ghanavati

**Affiliations:** 1 Department of Emergency Medicine, Imam Khomeini General Hospital, Ahvaz Jundishapur University of Medical Sciences, Ahvaz, Iran.; 2 Road Trauma Research Center, Guilan University of Medical Sciences, Rasht, Iran.

**Keywords:** Central venous catheters, vascular access devices, ultrasonography, emergencies, catheterization

## Abstract

**Introduction:**

Determining the proper angle for inserting central venous catheter (CV line) is of great importance for decreasing the complications and increasing success rate. The present study was designed to determine the proper angle of needle insertion for internal jugular vein catheterization.

**Methods:**

In the present case series study, candidate patients for catheterization of the right internal jugular vein under guidance of ultrasonography were studied. At the time of proper placing of the catheter, photograph was taken and Auto Cad 2014 software was used to measure the angles of the needle in the sagittal and axial planes, as well as patient’s head rotation.

**Result:**

114 patients with the mean age of 56.96 ± 14.71 years were evaluated (68.4% male). The most common indications of catheterization were hemodialysis (55.3%) and shock state (24.6%). The mean angles of needle insertion were 102.15 ± 6.80 for axial plane, 36.21 ± 3.12 for sagittal plane and the mean head rotation angle was 40.49 ± 5.09.

**Conclusion:**

Based on the results of the present study it seems that CV line insertion under the angles 102.15 ± 6.80 degrees in the axial plane, 36.21 ± 3.12 in the sagittal plane and 40.49 ± 5.09 head rotation yield satisfactory results.

## Introduction:

Insertion of central venous catheter (CV line) is a common procedure to reach the central veins with the aim of monitoring the patients regarding hemodynamic status, measuring central venous pressure (CVP), fluid therapy, infusion of vasoactive agents, etc. (1, 2). CV line insertion may be done either with the guidance of anatomic landmarks or ultrasonography. One of the problems caused by this procedure is the risk of pneumothorax and arterial puncture, which usually occur due to improper selection of angle and depth of needle insertion. The angle for needle insertion in both methods is suggested to be 30 - 45 degrees. Yet, determining the angle, which minimizes the mentioned complications, is still needed (-). In a study, the proper needle tilt angle for internal jugular vein catheterization was shown to be 75 degrees and higher (6). The present study aimed to determine the proper angle of needle insertion for right internal jugular vein catheterization. 

## Methods:

*Study design and setting*

The present case series study was designed to determine the proper angle of CV line insertion in internal jugular vein. Patients admitted to Imam Khomeini and Golestan Hospitals, Ahvaz, Iran were included. Based on the principles of Helsinki Declaration, researchers adhered to confidentiality of patient information and used the information only for research purposes. The study was approved by the Ethical Committee of Ahvaz Jondi Shapour University of Medical Sciences, Ahvaz, Iran. Written informed consent was obtained from all participants.

*Participants*

The participants were patients in need for catheterization of the right internal jugular vein, who were free of any evident deformity in chest and neck. Unsuccessful insertion of catheter, change in hemodynamic status or occurrence of dysrhythmia during procedure, and not permitting frequent photography and venipuncture, were among the most important exclusion criteria. 

*Procedure*

**Figure 1:**
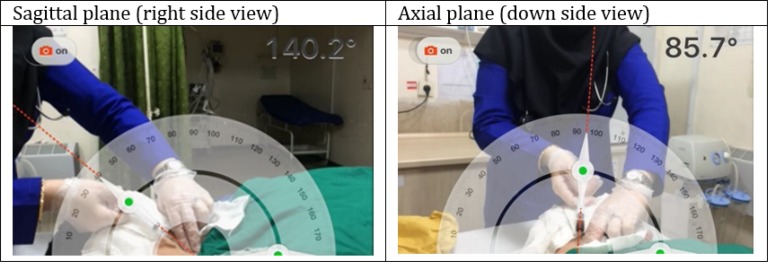
Sample photographs taken

After screening the cases in need of CV line insertion based on common standards (not being able to reach a peripheral vein, CVP monitoring, need to vasopressor agent, and …), patients underwent cardiopulmonary monitoring, pulse-oximetry, and nasal oxygen therapy. Then CV lines were inserted using the Seldinger method, under sterilized conditions, under ultrasonography guidance, and local anesthesia with lidocaine 1%. All patients were in supine or trendelenburg position. Sonography was done using a 7.5 MHz linear superficial probe under a sterilized nylon covering with a short axis approach on the right side of the neck in the anatomical position of internal jugular vein. Catheter needle was aimed at the apex of a triangle made from the crossing of the medial one-third of the clavicle bone in the base and 2 sternal and clavicular heads of sternocleidomastoid muscle towards the right nipple. After blood aspiration and ensuring the needle has entered the jugular vein, the needle and patient’s head position were kept fixed and photographs were taken from two perpendicular views, patient’s right side (sagittal plane) and patient’s down side (axial plane) (figure 1). The camera was equipped with a protractor application and the camera lens was in horizontal state. 

*Taking a photo from the sagittal plane:* While the patient was in supine position, the protractor was set to be parallel with the sagittal plane and when the needle entered the jugular vein and blood was aspirated, the center mark of the protractor was set at the place of needle insertion and the angle of needle with the horizontal line was measured. 

*Taking a photo from the axial plane:* While the patient was in supine position, the protractor was set to be parallel with the axial plane on the thoracic area and the center mark of the protractor was set at the place of needle insertion. Finally, when the needle entered the jugular vein and blood was aspirated, the angle of needle with horizontal line was measured. In addition, head rotation angle was measured in this plane by setting the zero point of the protractor on the mid sagittal line and measuring the angle of the line crossing the middle of the patient’s chin and the center mark of the protractor with vertical line.

Chest imaging was done to verify the place of the catheter and evaluate complications such as pneumothorax and hemothorax. All Imaging and photography was performed by a trained emergency resident and 2 nurses. The angles were measured using Auto Cad 2014 software. Demographic data of participants as well as duration of procedure and measured angles were collected via a predesigned checklist. In the end, data were presented as mean ± standard deviation and frequency and percentage using SPSS version 20.

**Table 1:** Baseline characteristics of studied patients

**Variable **	
Age **(year)**	
** Mean **	56.96 ± 14.71
** Range **	19 - 88
Sex **number (%)**	
** Male **	78 (68.4)
** Female **	36 (31.6)
Body mass index	
** > 30**	102 (89.5)
** < 30**	12 (10.5)
Cause of catheterization	
** Shock **	28 (24.6)
** Post resuscitation**	13 (11.4)
** Hemodialysis**	63 (55.3)
** Inaccessible peripheral vein**	8 (7)
** Catheter change**	1 (0.9)
** CVP measurement**	1 (0.9)
Number of performers	
** 1**	103 (90.4)
** 2**	11 (9.6)
Duration of catheterization **(minutes)**	
** Mean **	18.73 ± 1.51
** Range**	15 - 23

CVP: central venous pressure.

**Table 2:** Measured angles of head and needle in sagittal and axial planes

**Angles **	**Mean ** **±** ** SD (range)**
**Needle tilt ** **(degree)**	
In sagittal plane	36.21 ± 3.12 (28-45)
In axial plane	102.15 ± 6.80 (90-115)
**Head rotation (degree)**	
In axial plane	40.49 ± 5.09 (29-52)

SD: standard deviation.

## Results:

120 patients were evaluated, 6 of which were excluded due to complications such as hematoma (4 cases) and carotid artery perforation (2 cases). The mean age of participants was 56.96 ± 14.71 years (68.4% male). The most common indications of catheterization were dialysis (55.3%) and shock state (24.4%). Table 1 depicts the baseline characteristics of studied patients. Table 2 shows the measured angles of head and needle in sagittal and axial planes.

## Discussion:

The findings of the present study showed that CV line insertion under the angles 102.15 ± 6.80 degrees in the axial plane, 36.21 ± 3.12 in the sagittal plane, and 40.49 ± 5.09 head rotation may yield good results. 

In recent years, the number of patients in need of CV line insertion has increased due to various reasons (7). Arterial puncture, hematoma, pneumothorax, hemothorax, nerve injury, arrhythmia, air embolism, cardiac tear, tamponade, and probability of infection after surgery, are among the most important complications of catheterization (8). Catheterization, based on superficial landmarks is done in 1 attempt in about 20% of the time, while with the help of bedside ultrasonography, catheterization can be done with fewer complications and higher success rate (8). 

In a study aiming to determine the proper angle of needle insertion in the sagittal plane on the internal jugular vein, it was determined that a 16 degree insertion angle from the sagittal plane results in an increase in the probability of successful catheterization (9). In our study, mean sagittal angle of needle tilt from the horizontal line was about 36 degrees, which is vastly different from the mentioned study. In another study, the best angle for head rotation in internal jugular vein catheterization was reported to be ≥ 75 degrees (6). Head rotation in the present study was found to be 40 degrees from vertical line. To date, few studies have been carried out for determining the accurate axial angle of the needle. Considering the importance of this matter, it is suggested to design studies with proper sample size calculated based on existing data in the future.

## Conclusion:

Based on the results of the present study it seems that CV line insertion under the angles 102.15 ± 6.80 degrees in the axial plane, 36.21 ± 3.12 in the sagittal plane and 40.49 ± 5.09 head rotation yield satisfactory results. 
